# Supplementary material for: The Paradoxical Effect Hypothesis of Abused Drugs in a Rat Model of Chronic Morphine Administration
Source: J Clin Med. 2021 Jul 21;10(15):3197. doi: 10.3390/jcm10153197 (PMC8348660; doi:10.3390/jcm10153197)
Supplement: Supplementary file 1 [file jcm-10-03197-s001.zip › jcm-1277506-supplementary.pdf]

Supplementary Materials

Supplementary Materials and Methods

Animals

For this study, 202 male Wistar rats (weighing 250-300 g at the beginning of the experiment; BioLasco Taiwan Co., Ltd.) were housed in a colony room, with a pair of rats in each plastic home cage (47 cm long × 26 cm wide × 21 cm high) and with hardwood laboratory bedding (Beta Chip). The colony room was kept at a constant temperature (approximately 23ºC ± 2ºC) with a 12 h/12 h light/dark cycle (lights on: 6:00-18:00). Water and food chow were freely offered. However, the rats were deprived of water during specific phases. All experiments were performed in compliance with the Animal Scientific Procedures Act of 1986 and received approval from the Institutional Animal Care and Use Committee (IACUC) of Fo Guang University (protocol #: 101002). Every effort was made to minimize animal suffering and to limit the number of animals used.

Apparatus

Lickometer. The lickometer device included a white panel, a wire-mesh cage, and a 25 ml burette with 0.1 ml graduation. The burette was joined to the white panel and mounted in front of the wire-mesh cage. The analysis in the present study only included the intake volume of the saccharin solution.

CPP apparatus. The CPP task was a T-shaped apparatus with three wooden compartments. This task had two distinct, approximately square compartments (49 × 42 × 35 cm high) and an intermediary shuttle compartment (33 × 16 × 16 cm high). The wood partitions separated three compartments: two preference compartments and one shuttle compartment. Through the shuttle compartment, the other two preference compartments were accessible. A transparent acrylic glass observation wall was on the

front side of the CPP task for each preference compartment. One compartment had black-and-white horizontal-striped walls with a wire-grid floor. The other of the preference compartments was painted white with a wood-chip bedding floor.

Statistical analysis

In Experiment 1, the volume of saccharin solution intake was analyzed by 5 × 5 mixed two-way ANOVA for CTA. If need be, Tukey's honestly significant difference post hoc test was carried out for each session. For CPP, the mean spent time was analyzed by a dependent t-test for drug-unpaired and drug-paired sides among 0-40 mg/kg morphine groups. A p value less than 0.05 was considered significant in all cases.

In Experiment 2, the intake volume of the saccharin solution was measured as the CTA index. The CTA responses in the conditioning and extinction phases were analyzed using a 2 × 5 mixed two-way ANOVA. An independent t-test was conducted for the reinstatement phase to analyze the intake volume of the 0.1% saccharin solution. When appropriate, post hoc with Tukey's honestly significant difference was performed for each session. A p value less than 0.05 was considered significant in all cases. A one-way ANOVA analyzed the immunohistochemical staining data for c-Fos and p-ERK expressions for the saline and morphine groups to analyze the specific brain areas in the conditioning, extinction, and reinstatement phases. The plasma corticosterone levels were analyzed using a one-way ANOVA for the saline and morphine groups at baseline and in the conditioning, extinction, and reinstatement phases. To test homeostasis of neural substrates for the brain, c-Fos and p-ERK expressions were respectively analyzed by a 3 × 2 × 12 mixed (Phase × Drug × Neural Substrate) three-way ANOVA. Furthermore, a 2 × 12 mixed (Drug × Neural

Substrate) two-way ANOVA and one-way repeated trend analysis were conducted in the conditioning, extinction, and reinstatement phases for saline and morphine groups to analyze the trend-line, respectively. Trends are identified by drawing lines, called trend lines. The different orders of trend lines indicate the different shapes of trends, such as one order trend-line for linear, two orders trend-line for quadratic, three orders trend-line for cubic, etc. Finally, a one-way ANOVA was analyzed for the saline and morphine groups for selected brain areas. Values of $p < 0.05$ were considered statistically significant. In addition, numbers and percentages of neural substrates were analyzed for c-Fos or p-ERK expression on conditioning, extinction, and reinstatement phases after morphine-induced conditioned suppression of saccharin solution intake.

Review

In discussion, the present study used the Pubmed database to review publications related to morphine addiction for reward and aversion tasks during 1970-2019.

Figure S1.

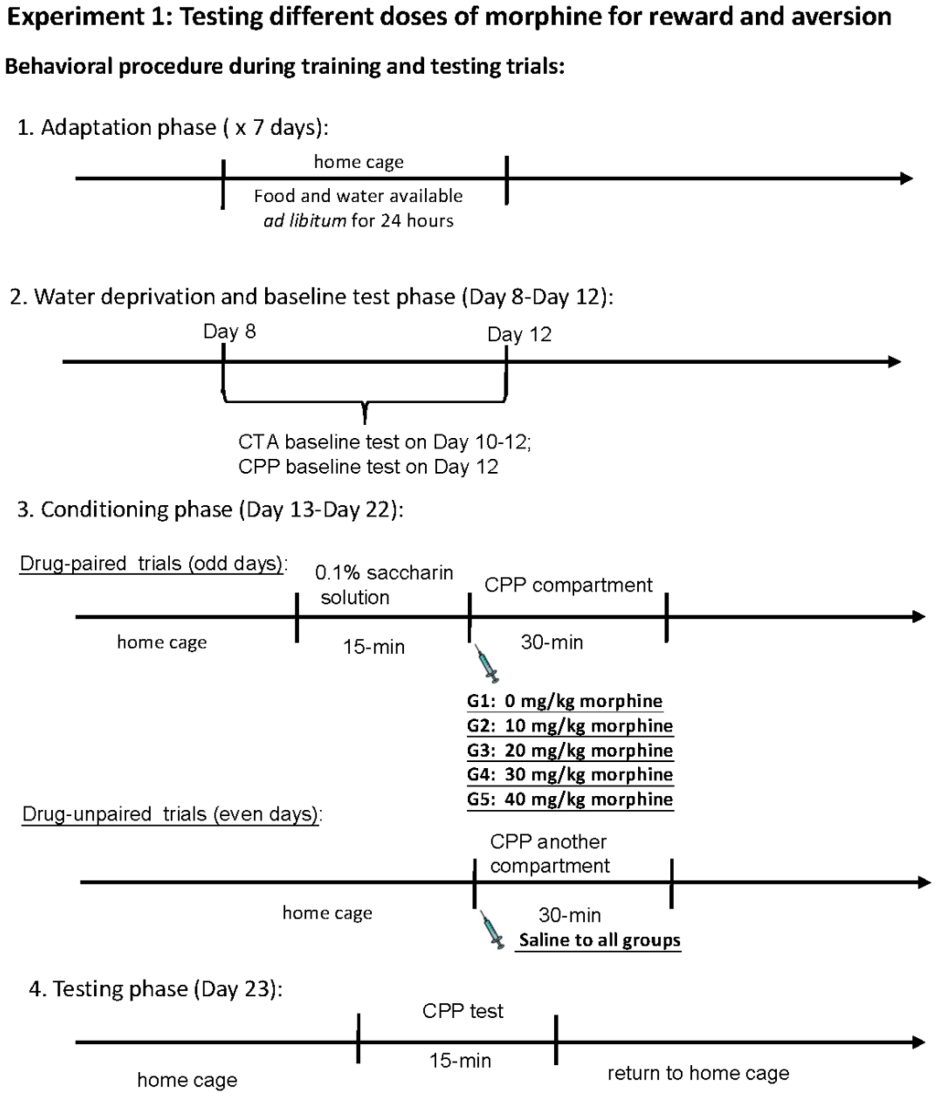

Figure S1. Overview of the experimental procedures for Experiment 1. The behavioral procedures during the adaptation, water deprivation and baseline test, conditioning, and testing phases are shown in detail for conditioned taste aversion (CTA) and conditioned place preference (CPP). On conditioning, Experiment 1 shows how various doses of morphine (0, 10, 20, 30, and 40 mg/kg) were paired with 0.1% saccharin solution CS1 and then injected alongside morphine US to form CTA. Then, the rat with the morphine injection was placed in one context of the CPP, CS2, to produce CPP in the drug-paired trial on even-numbered days. On odd-numbered days, the rat stayed in the home cage without the CTA procedure. All rats were then injected

with saline in another context of CPP for 30 min. Finally, all rats were tested in the CPP box for 15 min. The dose of 10 mg/kg morphine was used to determine the most suitable aversive dose of morphine for use in Experiment 2.

Figure S2

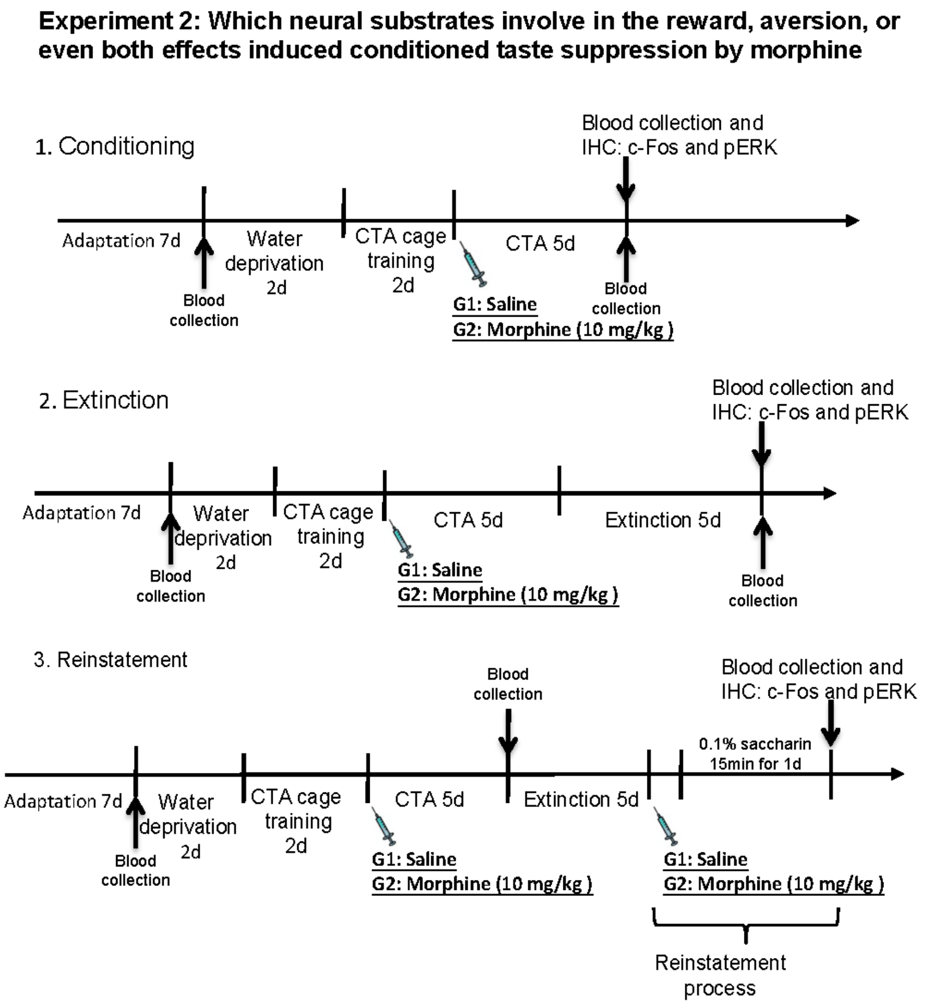

Figure S2. Overview of the experimental procedures for Experiment 2. The behavioral procedure and immunohistochemical staining with c-Fos or p-ERK were conducted during the stages of conditioning, extinction, reinstatement.

Figure S3

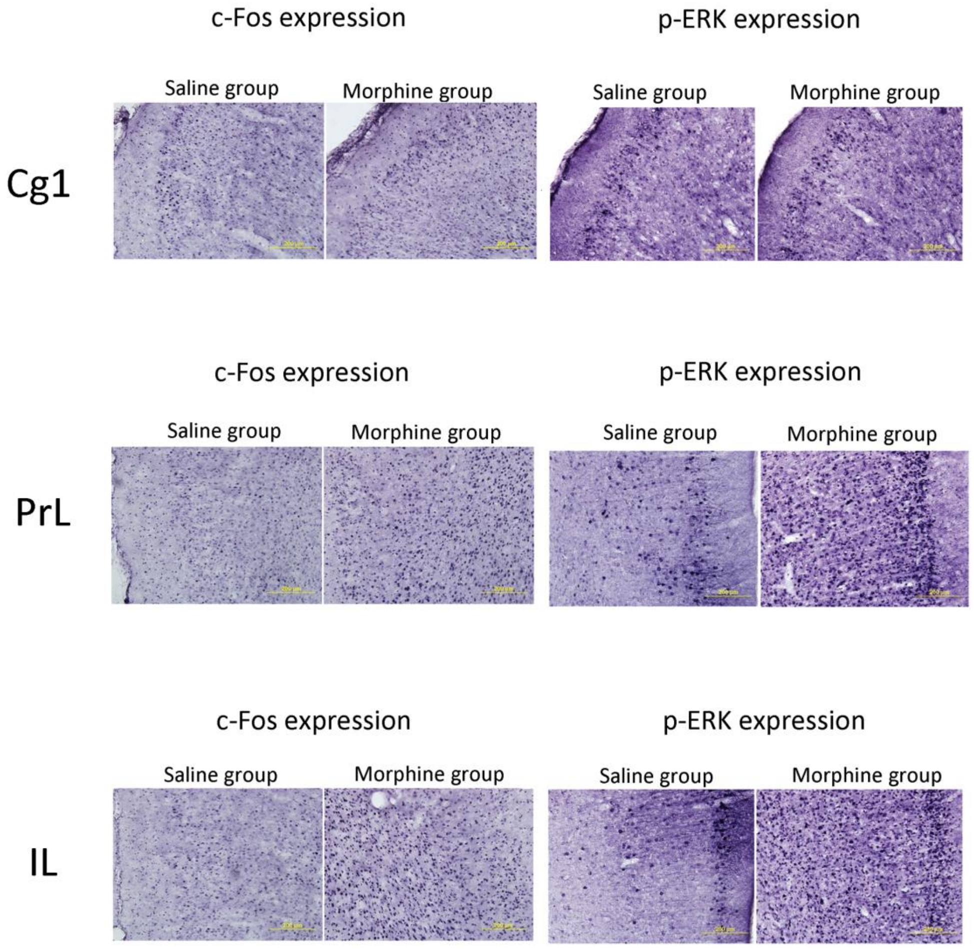

Figure S3. Representative photomicrographs of c-Fos and p-ERK immunoreactivity for the Cg1, PrL, and IL in the saline and morphine groups during conditioning. Scale bar represents 200 μm.

Figure S4.

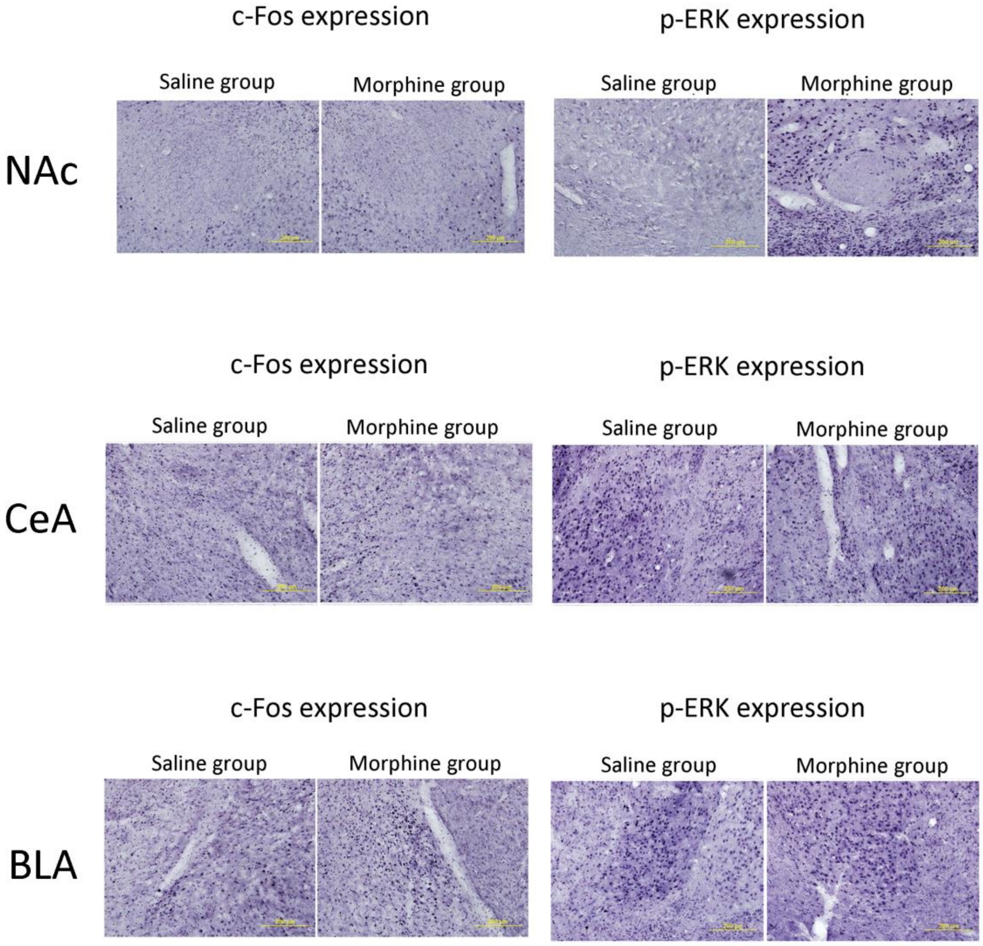

Figure S4. Representative photomicrographs of c-Fos and p-ERK immunoreactivity for the NaC, CeA, and BLA in the saline and morphine groups during conditioning. Scale bar represents 200 µm.

Figure S5

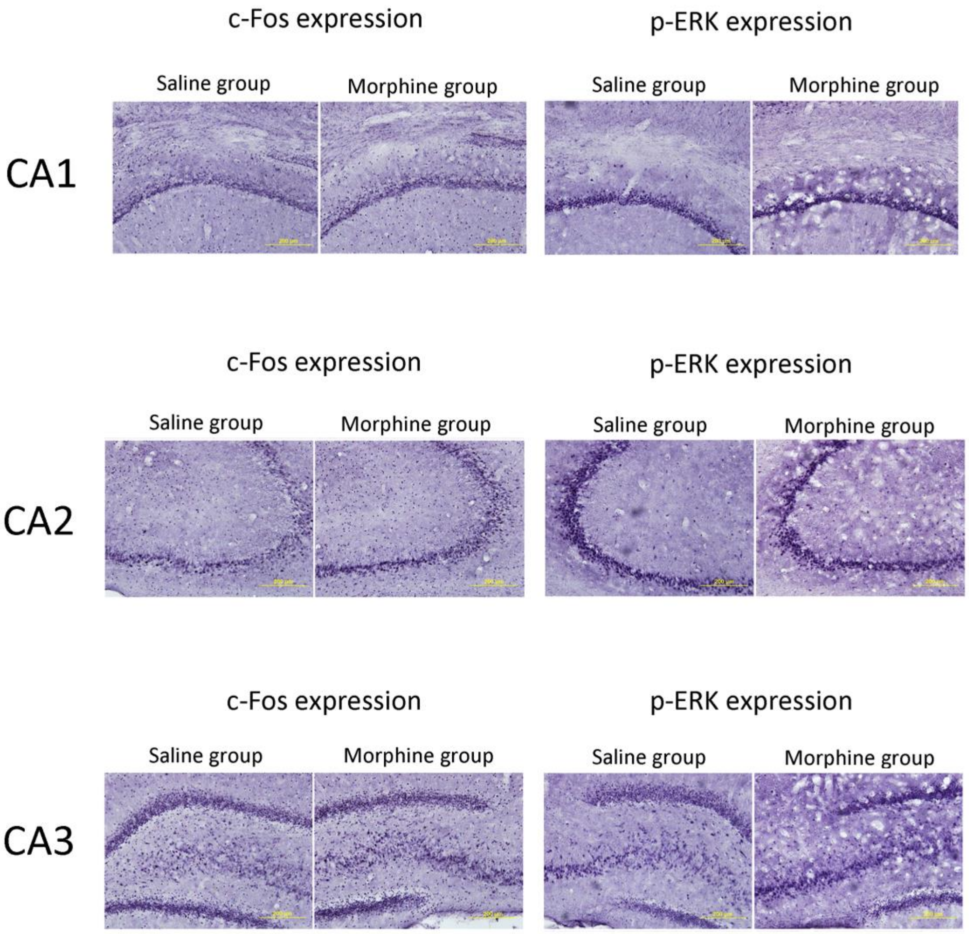

Figure S5. Representative photomicrographs of c-Fos and p-ERK immunoreactivity for the CA1, CA2, and CA3 in the saline and morphine groups during conditioning. Scale bar represents 200 µm.

Figure S6

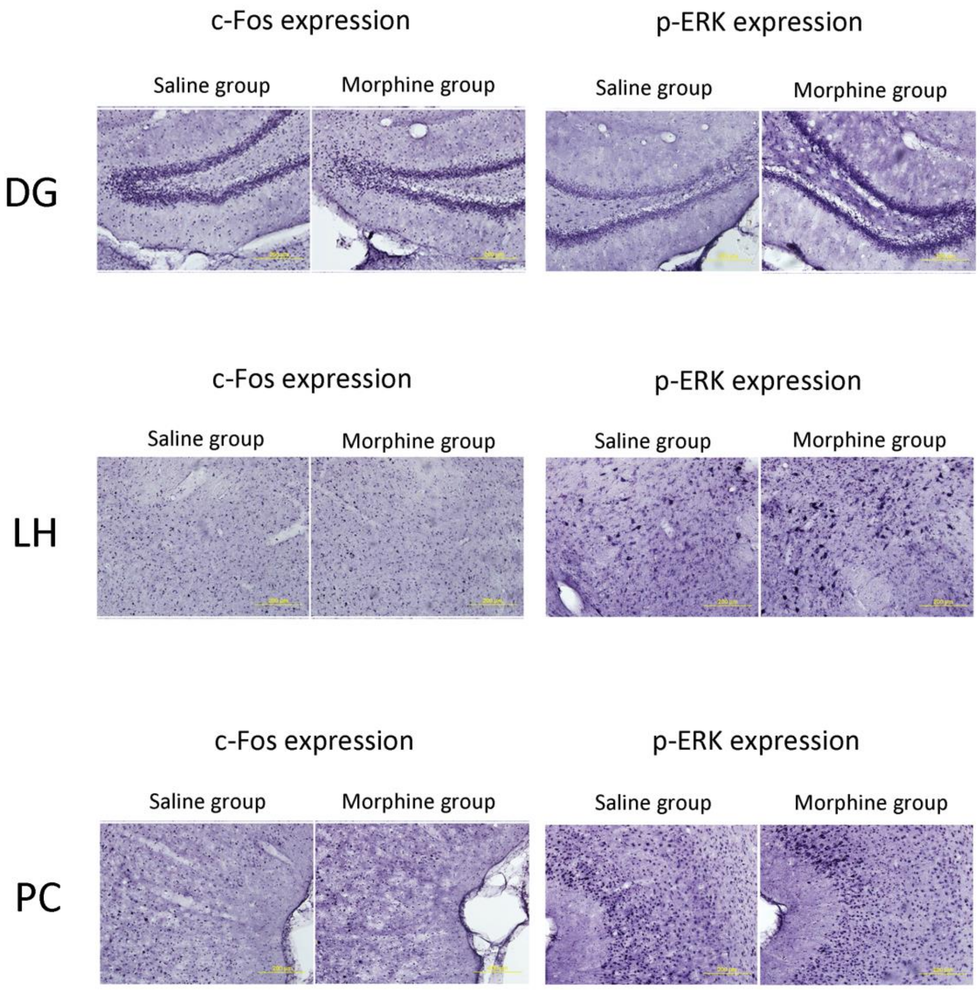

Figure S6. Representative photomicrographs of c-Fos and p-ERK immunoreactivity for the DG, LH, and PC in the saline and morphine groups during conditioning. Scale bar represents 200 µm.

Figure S7

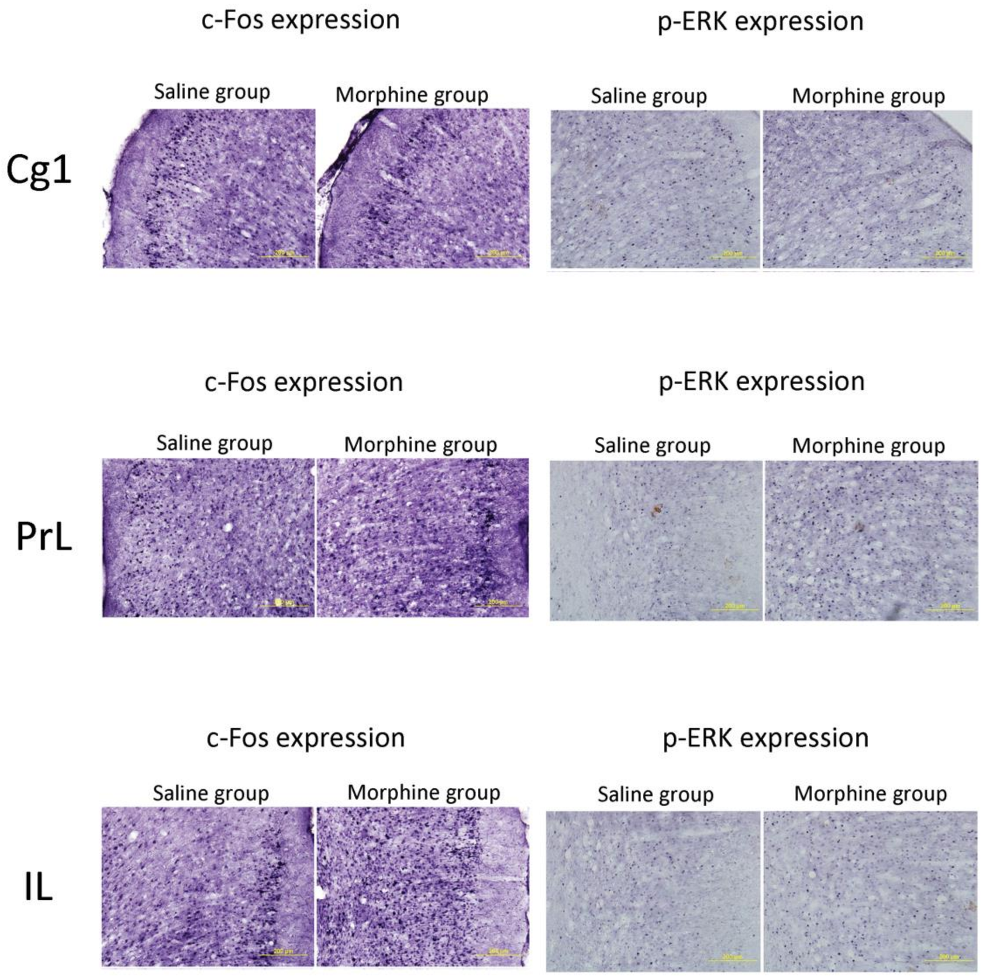

Figure S7. Representative photomicrographs of c-Fos and p-ERK immunoreactivity for the Cg1, PrL, and IL in the saline and morphine groups during extinction. Scale bar represents 200 µm.

Figure S8

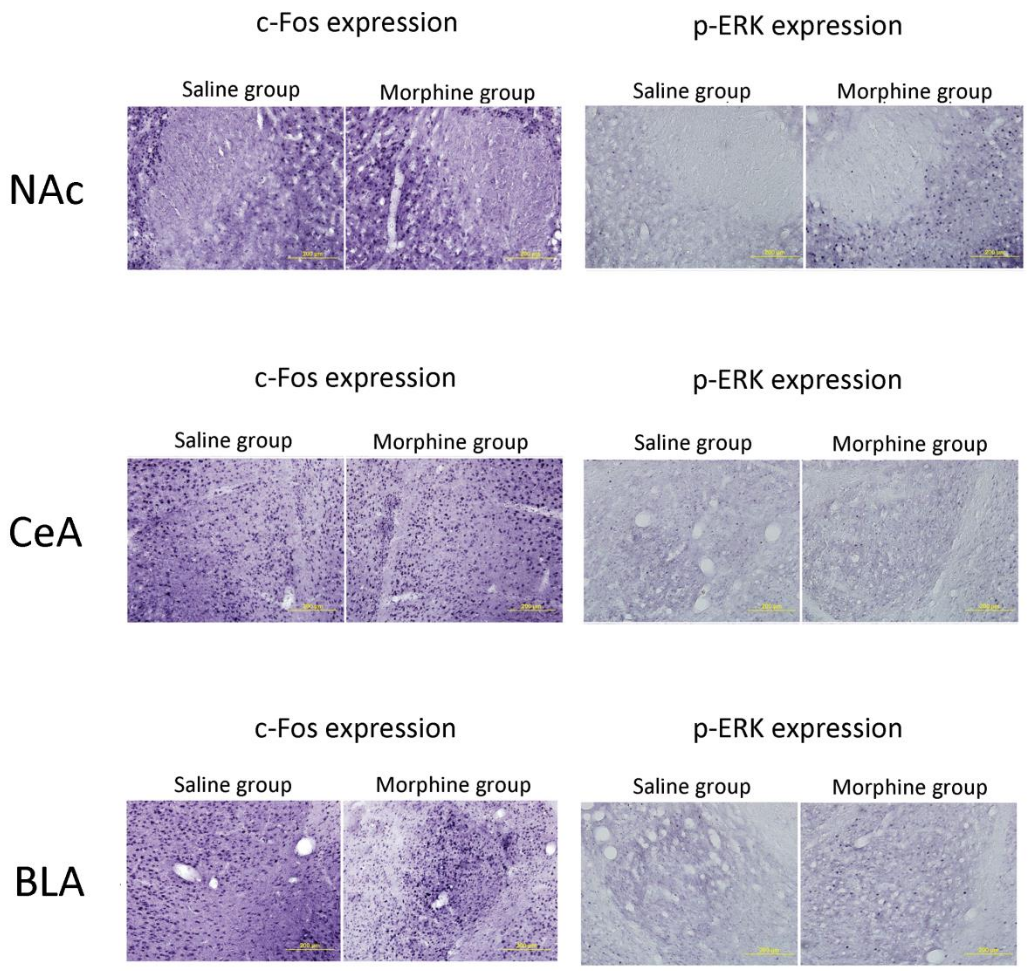

Figure S8. Representative photomicrographs of c-Fos and p-ERK immunoreactivity for the NaC, CeA, and BLA in the saline and morphine groups during extinction. Scale bar represents 200 µm.

Figure S9

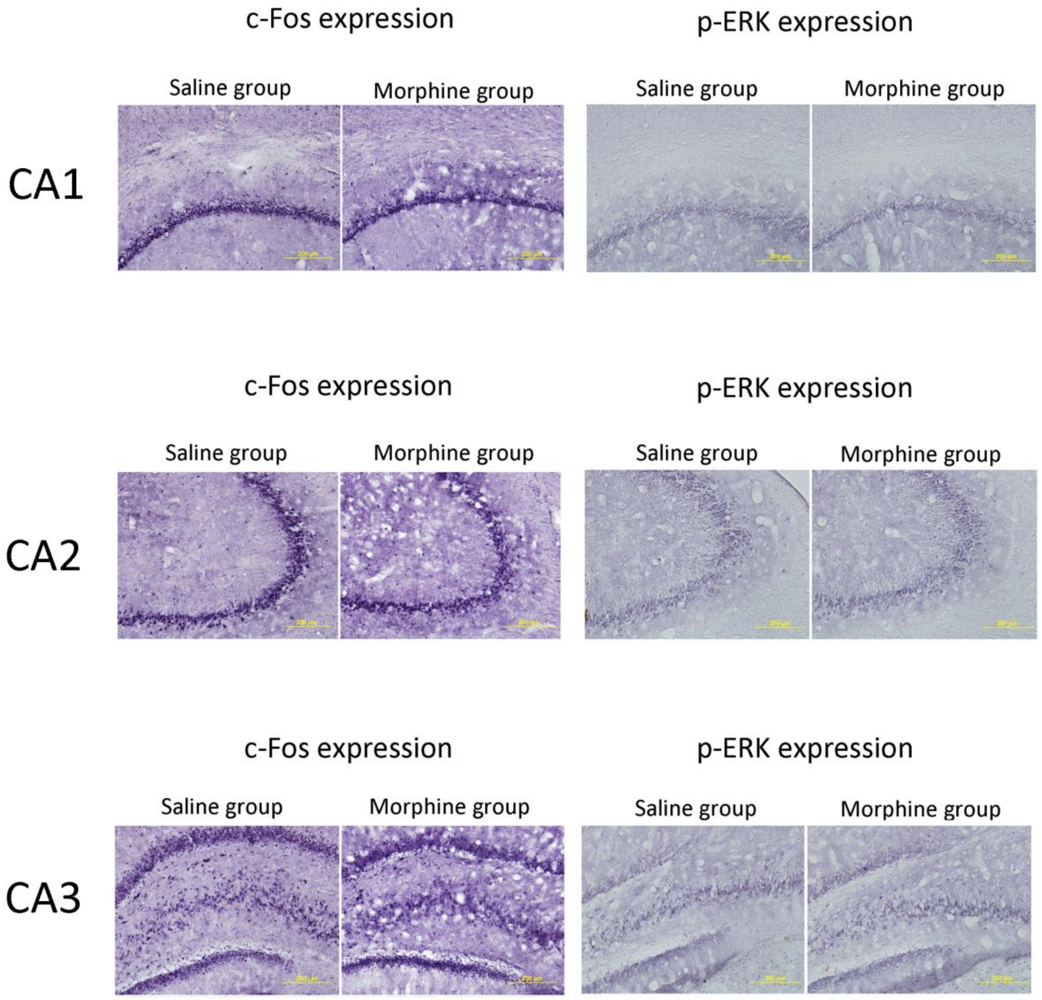

Figure S9. Representative photomicrographs of c-Fos and p-ERK immunoreactivity for the CA1, CA2, and CA3 in the saline and morphine groups during extinction. Scale bar represents 200 µm.

Figure S10

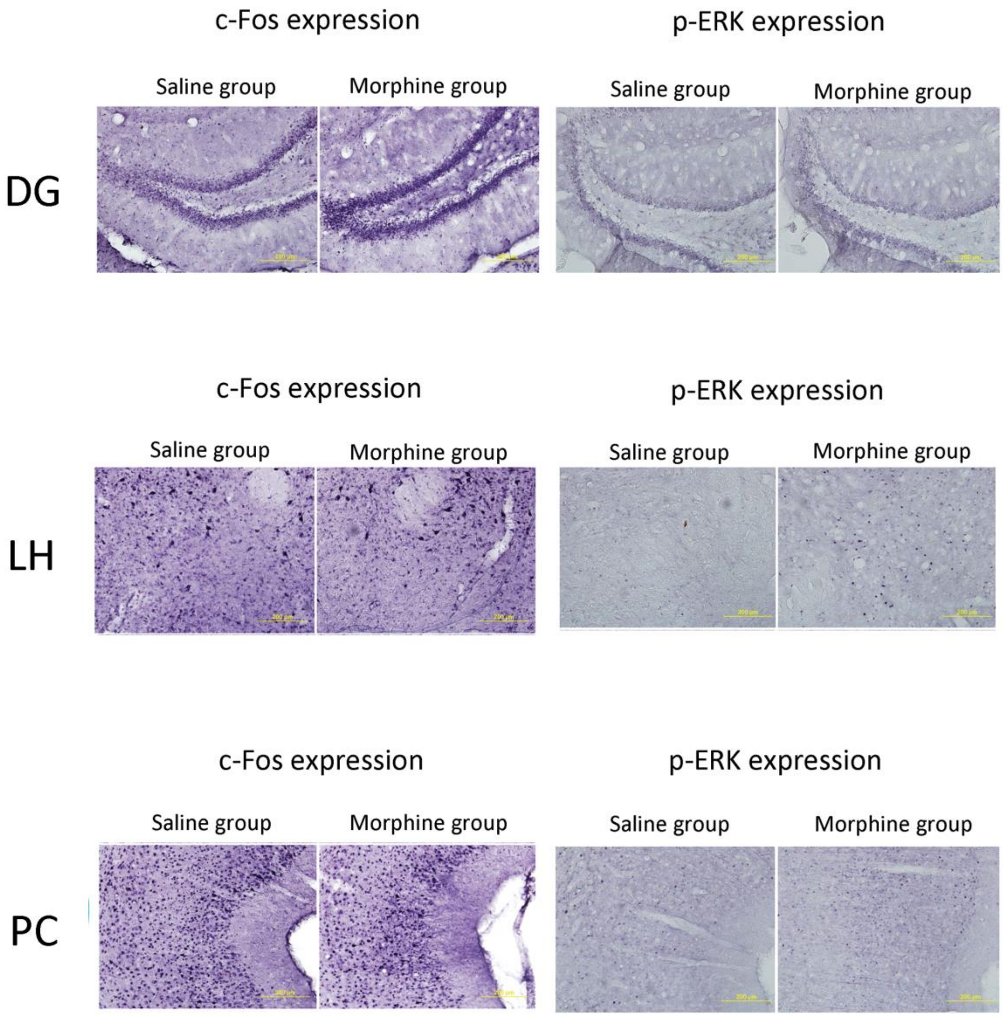

Figure S10. Representative photomicrographs of c-Fos and p-ERK immunoreactivity for the DG, LH, and PC in the saline and morphine groups during extinction. Scale bar represents 200 µm.

Figure S11

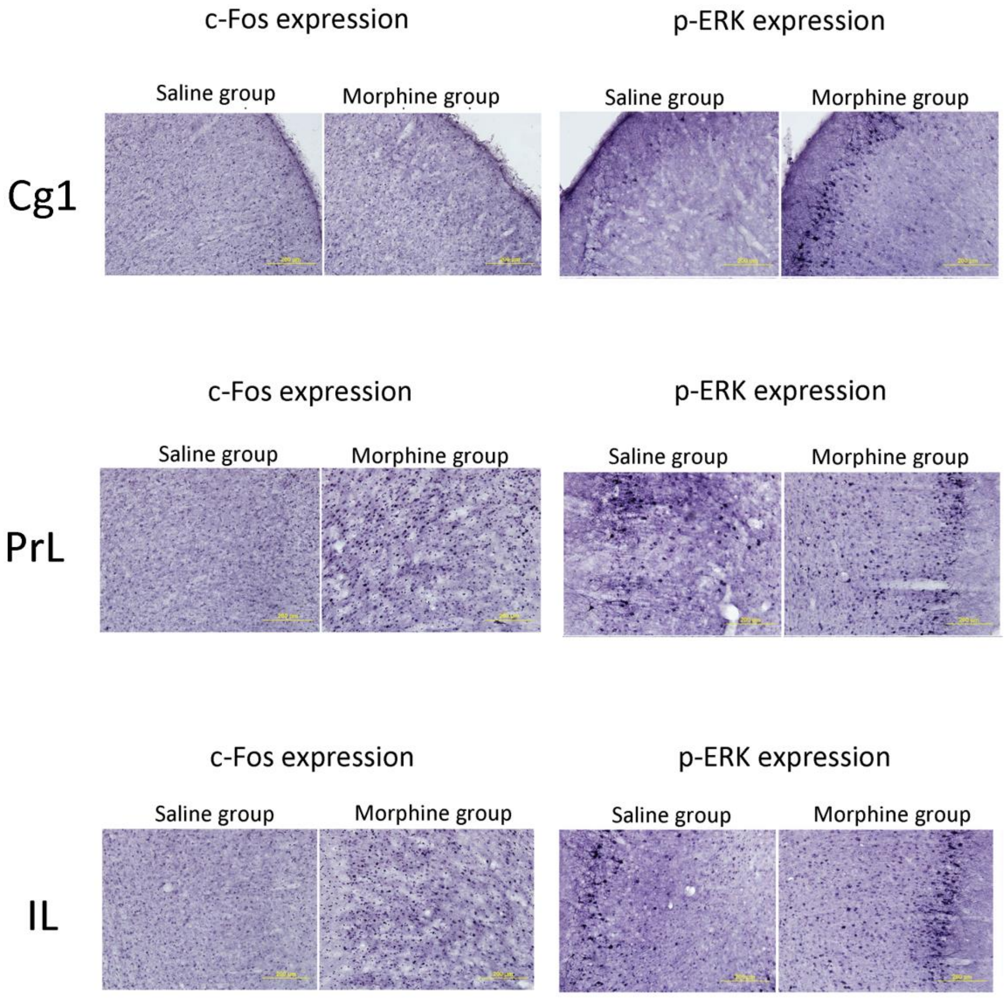

Figure S11. Representative photomicrographs of c-Fos and p-ERK immunoreactivity for the Cg1, PrL, and IL in the saline and morphine groups during reinstatement. Scale bar represents 200 μm.

Figure S12.

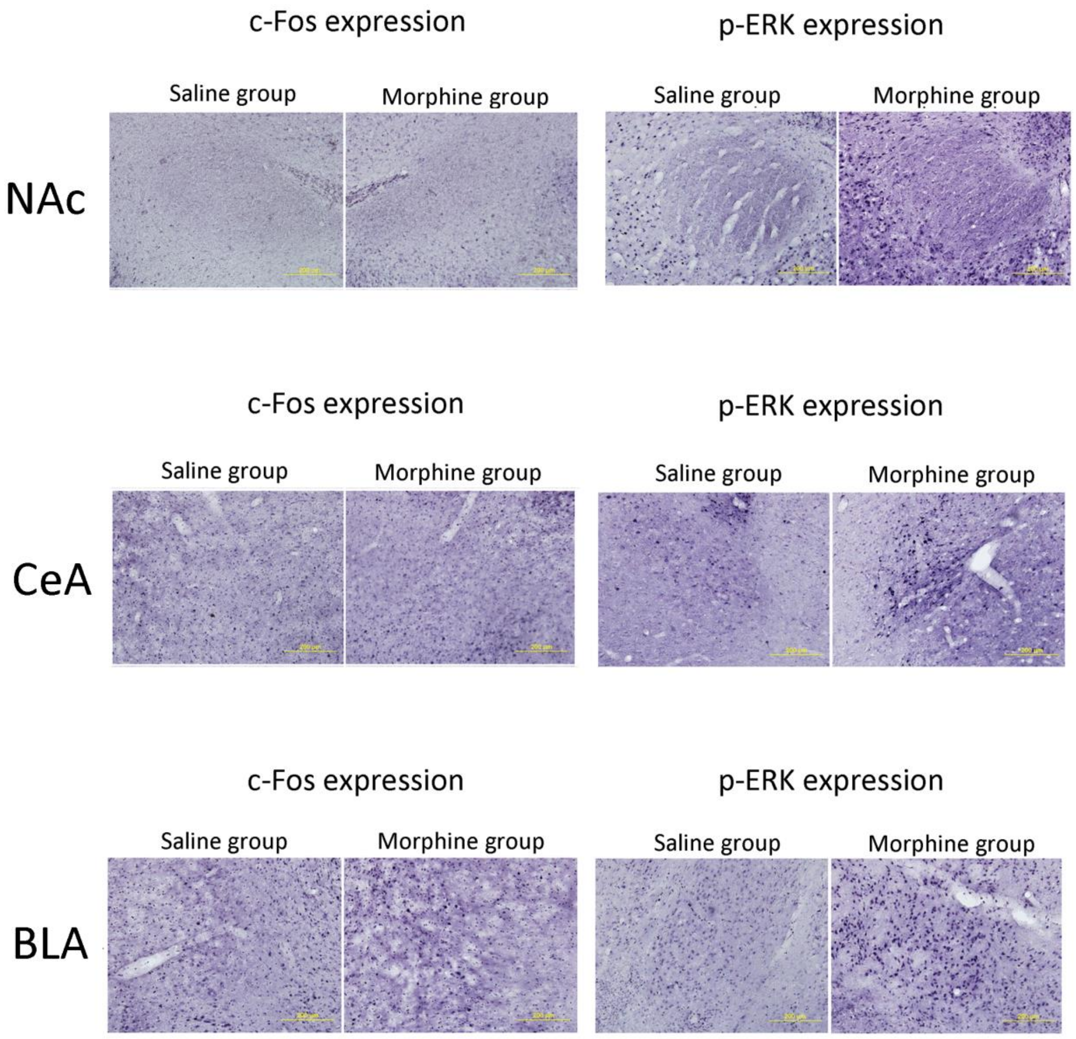

Figure S12. Representative photomicrographs of c-Fos and p-ERK immunoreactivity for the NaC, CeA, and BLA in the saline and morphine groups during reinstatement. Scale bar represents 200 µm.

Figure S13

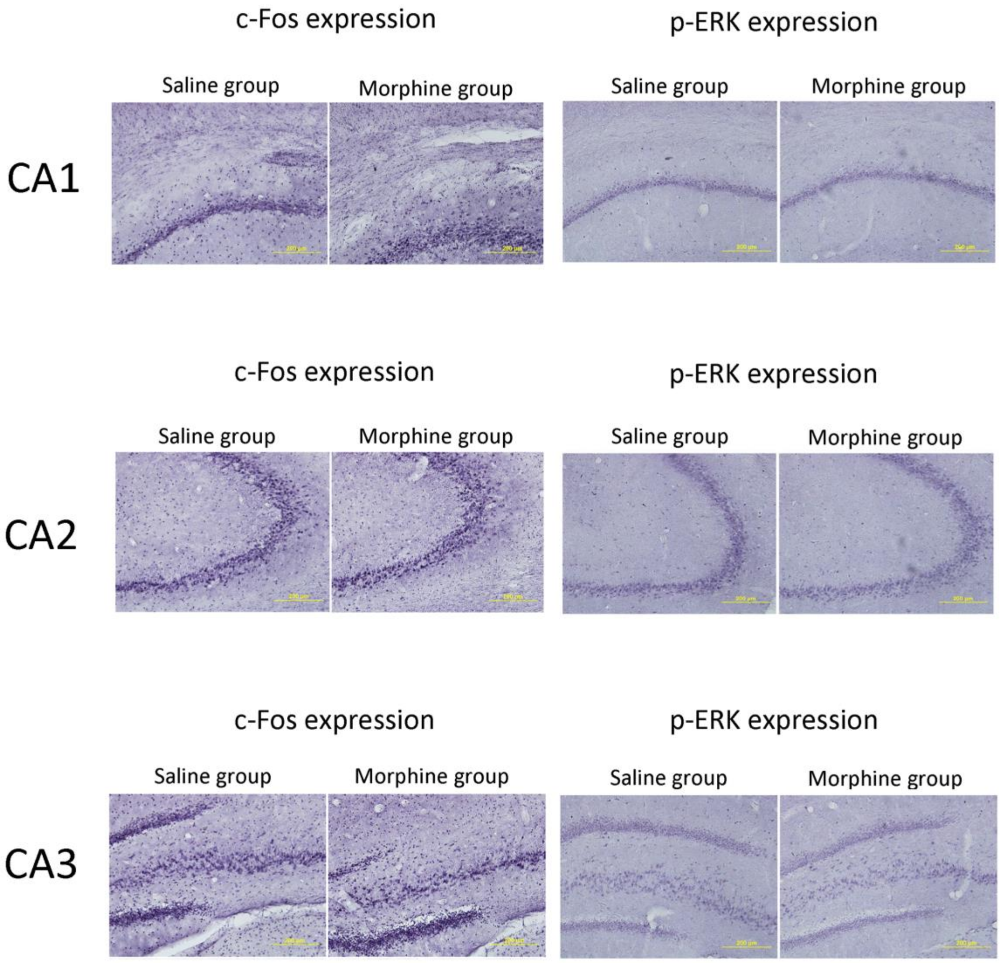

Figure S13. Representative photomicrographs of c-Fos and p-ERK immunoreactivity for the CA1, CA2, and CA3 in the saline and morphine groups during reinstatement. Scale bar represents 200 µm.

Figure S14

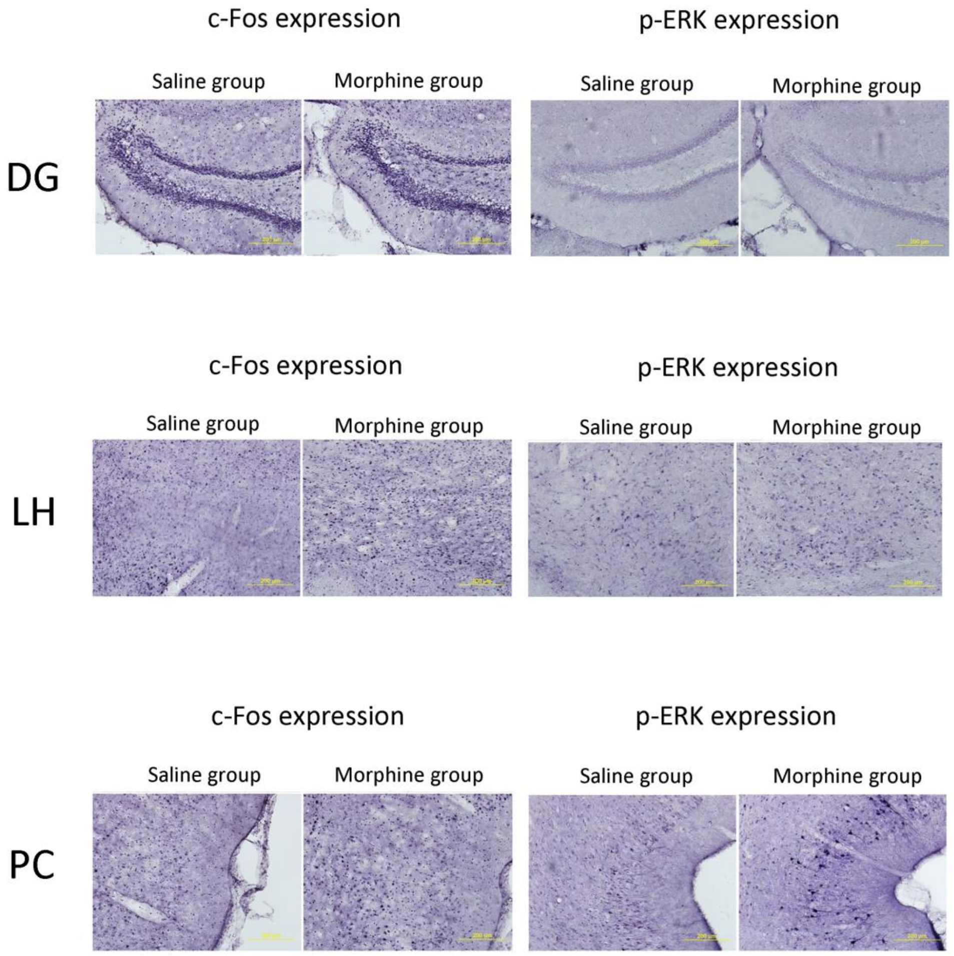

Figure S14. Representative photomicrographs of c-Fos and p-ERK immunoreactivity for the DG, LH, and PC in the saline and morphine groups during reinstatement. Scale bar represents 200 µm.
